# Longitudinal Epidemiology of Coagulase-Positive Staphylococcal Species Recovered from Humans, Pets, and Household Environments

**DOI:** 10.3390/antibiotics15060552

**Published:** 2026-05-30

**Authors:** Gwen L. Wardenburg, Alaina L. Robinson, Lisa M. Richardson, Mary G. Boyle, Carol M. Kao, Eleanor S. Archer, Carey-Ann D. Burnham, Stephanie A. Fritz

**Affiliations:** 1 Departments of Pediatrics, Washington University School of Medicine, St. Louis, MO 63110, USA; 2 Departments of Molecular Microbiology, Washington University School of Medicine, St. Louis, MO 63110, USA; 3 Departments of Pathology & Immunology, Washington University School of Medicine, St. Louis, MO 63110, USA; 4 Departments of Medicine, Washington University School of Medicine, St. Louis, MO 63110, USA

**Keywords:** *Staphylococcus aureus*, methicillin-resistant *Staphylococcus aureus* (MRSA), *Staphylococcus pseudintermedius*, *Staphylococcus schleiferi*, *Staphylococcus intermedius* group, veterinary, companion animal, pet, colonization, One Health

## Abstract

**Background:** Veterinary staphylococcal species, including the *Staphylococcus intermedius* group (SIG) and *Staphylococcus schleiferi*, colonize and infect companion animals (pets) and humans. This study investigated the longitudinal colonization prevalence of veterinary staphylococci among pets, their humans, and household environments to identify factors associated with carriage and to characterize antibiotic susceptibility trends. **Methods:** Children with community-onset *Staphylococcus aureus* skin and soft tissue infection (SSTI), their household contacts, and pets were enrolled in the “*Staph* Hygiene Intervention for Eradication (SHINE)” trial. At five study visits over 9 months, humans, pets, and household surfaces were swabbed for staphylococcal species detection and health information was collected. **Results:** The 104 households containing pets comprised 459 humans and 178 pets (136 dogs and 42 cats). Veterinary staphylococci were recovered from 110 pets (62%), 39 (9%) humans, and environmental surfaces in 55 (53%) households. SIG was the most commonly recovered veterinary staphylococci. Ninety percent of colonized humans were colonized with the same staphylococcal species as their pet. In multivariable analyses, dogs were more likely to be colonized than cats and a higher burden of environmental surface contamination was associated with pet and human colonization. The prevalence of methicillin-resistant veterinary staphylococci was low, but resistance to multiple other antibiotics was common among these methicillin-resistant isolates. **Conclusions:** Carriage of the same staphylococcal species and temporal colonization patterns between companion animals and their owners may suggest cross-species sharing, with the environment serving as a reservoir.

## 1. Introduction

Coagulase-positive staphylococci, including *Staphylococcus aureus*, the *Staphylococcus intermedius* group (SIG, comprising *Staphylococcus pseudintermedius, Staphylococcus intermedius*, and *Staphylococcus delphini*), and *Staphylococcus schleiferi*, pose significant health concerns to human and veterinary medicine [1,2]. While these staphylococcal species are frequently commensals, they are also important pathogens. The SIG organisms, as well as *Staphylococcus schleiferi*, (hereafter referred to collectively as “veterinary staphylococci”) have the ability to colonize and infect small animals [3]. These veterinary staphylococci cause surgical-site infections, superficial and deep pyoderma, otitis externa, and urinary tract infections in companion animals [4,5,6,7,8]. There is increasing evidence that these species are also important pathogens in humans, especially those living with a companion animal or working in the veterinary medicine field [9,10,11,12]. In humans, veterinary staphylococci have been reported to cause SSTIs, rhinosinusitis, joint and bone infections, pneumonia, bacteremia, endocarditis, and device-related infections [9,10,13,14,15,16,17,18,19,20,21]. With advances in clinical microbiology, specifically the implementation of matrix-assisted laser desorption ionization time-of-flight (MALDI-TOF) mass spectrometry, it has come to light that many human infections, previously classified as *S. aureus*, were actually caused by SIG species, thus underestimating the incidence of these infections [10,22]. In humans, *S. aureus* is the leading cause of skin and soft tissue infections (SSTI) and also causes severe, invasive infections [23]. *S. aureus* has also been recovered from companion animals (especially those living with humans colonized or infected with *S. aureus*) and can cause SSTIs and surgical-site infections in these companion animals [3,24,25,26,27].

To date, the epidemiology of veterinary staphylococci in companion animals and humans remains understudied. Households have a unique risk of transmission of veterinary staphylococci and *S. aureus* due to repetitive close contact between humans, companion animals, and environmental surfaces [28]. Indeed, staphylococcal species are known for their ability to persistently contaminate environmental surfaces; veterinary staphylococci have been recovered most commonly from surfaces associated with companion animals, such as pet beds and feeding bowls [6,11,29,30]. Importantly, increasing prevalence of antimicrobial-resistant strains of *S. aureus*, SIG, and *S. schleiferi* may pose additional burdens for clinicians [9,13]. Currently, there is a lack of understanding regarding the factors associated with the carriage of these species in companion animals and humans [3,9,27,31,32,33,34].

The primary objective of this study was to determine the longitudinal colonization prevalence and species concordance of veterinary staphylococci among companion animals, their humans, and their environments within households of children with a history of *S. aureus* SSTI. Additionally, this study aimed to identify factors associated with animal and human carriage and to characterize antibiotic susceptibility trends among these veterinary staphylococcal species.

## 2. Results

### 2.1. Study Population Characteristics

**Figure 1:**
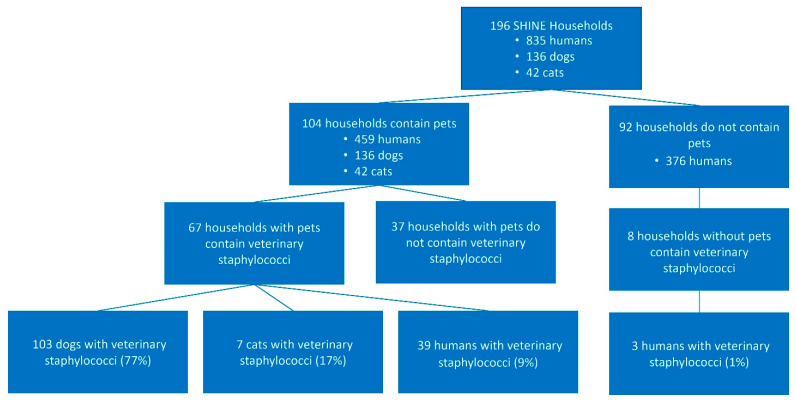
Colonization prevalence expressed as percent of dogs, cats, and humans colonized with veterinary staphylococci throughout the course of the 9-month study.

Of the 196 households participating in the SHINE trial, 104 had at least one indoor pet dog or cat. Within the cohort of 104 pet-containing households, there were 459 humans and 178 pets, including 136 dogs and 42 cats. Forty-eight percent of households had one pet, 35% had 2 pets, and 17% had 3 or more pets. The median number of pets per household was 2. Veterinary staphylococci colonization occurred in a pet, human, environmental surface, or a combination of the three, in 64% of households with pets and 9% of households that did not contain pets (Figure 1). Of these non-pet households, three humans from two households were colonized with veterinary staphylococci, and 17 total environmental surfaces were contaminated across six households. In these non-pet households, veterinary staphylococci carriage was only recovered at one study visit within each household and did not persist. Thus, the remainder of our analyses focused on households with pets.

### 2.2. Staphylococcal Species Carriage in Pets and Humans

#### 2.2.1. Pets

**Figure 2:**
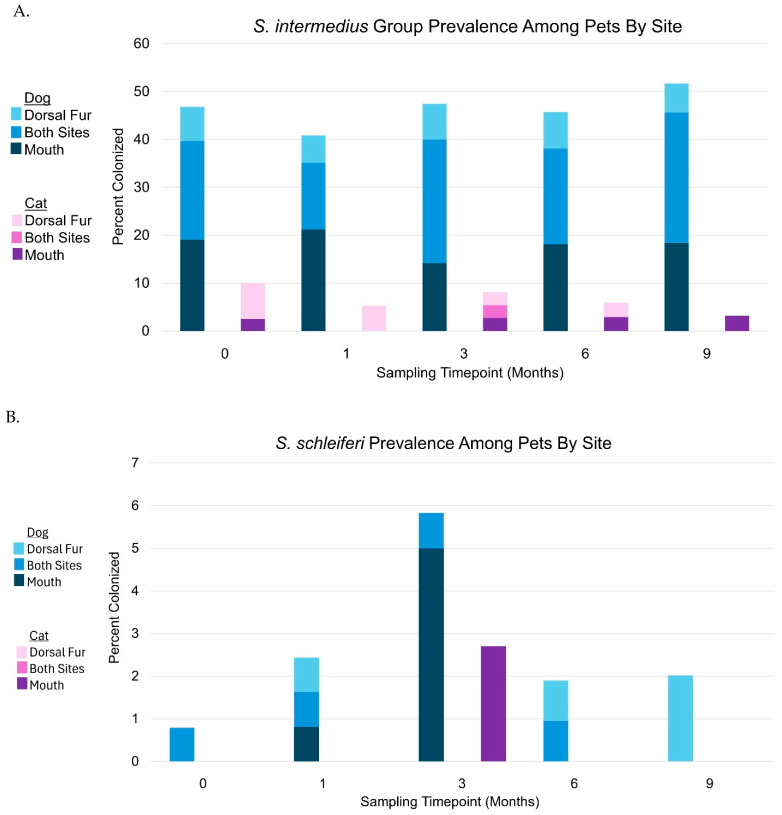

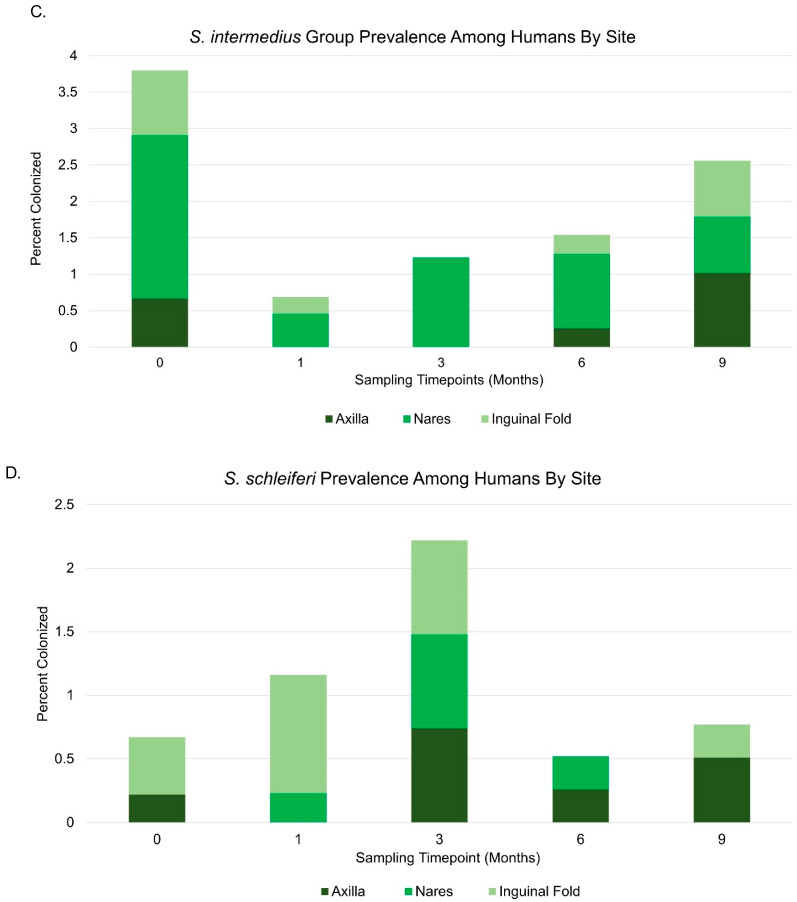
Longitudinal colonization patterns for *S. intermedius* group and *S. schleiferi* by anatomical sites for pets and humans. (**A**) Longitudinal pet colonization prevalence with *S. intermedius* group by anatomical site; (**B**) longitudinal pet colonization prevalence with *S. schleiferi* by anatomical site; (**C**) longitudinal human colonization prevalence with *S. intermedius* group by anatomical site; (**D**) longitudinal human colonization prevalence with *S. schleiferi* by anatomical site.

Of 178 pets enrolled in our study, 62% (110 total: dogs *n* = 103, cats *n* = 7) were colonized with veterinary staphylococci (Table 1). Ninety percent (dogs *n* = 94, cats *n* = 6) of colonized pets were found to be exclusively colonized with SIG. The remaining 10% (dogs *n* = 9, cats *n* = 1) of pets were colonized with both SIG and *S. schleiferi* at the same or different sampling timepoints longitudinally. Of the 110 pets colonized with veterinary staphylococci, 25% (dogs *n* = 24, cats *n* = 3) were colonized at one sampling timepoint, 28% (dogs *n* = 29, cats *n* = 2) were intermittently colonized, and 47% (dogs *n* = 50, cats *n* = 2) were persistently colonized (positive at two or more consecutive samplings). The mouth was the most frequent site of veterinary staphylococci carriage (Figure 2A,B).

*S. aureus* colonization occurred in 56 out of 178 pets (31%) (dogs *n* = 45, cats *n* = 11). Of these, 66% (dogs *n* = 29, cats *n* = 8) were colonized with *S. aureus* at one sampling timepoint, 16% (dogs *n* = 8, cats *n* = 1) were intermittently colonized, and 18% (dogs *n* = 8 and cats *n* = 2) were persistently colonized. The mouth was the most common site of *S. aureus* carriage in pets.

Nineteen dogs were colonized with veterinary staphylococcal species and *S. aureus* at the same body site and timepoint (Table 2). There were no cats colonized with veterinary staphylococci and *S. aureus* at the same body site and timepoint.

#### 2.2.2. Humans

Veterinary staphylococci carriage was found in 39 out of 459 (9%) humans living in a household with a pet (Table 1). Seventy-nine percent of humans were colonized with SIG, 15% were colonized with *S. schleiferi*, and 5% were colonized with both SIG and *S. schleiferi* at different timepoints and/or body sites. The nares (42%) were the most frequently colonized body site (Figure 2C,D). Colonization with the same veterinary staphylococcal species at two or more body sites at the same sampling timepoint occurred in 23% of colonized humans. In 19 (76%) households, only one household member was colonized.

**Figure 3:**
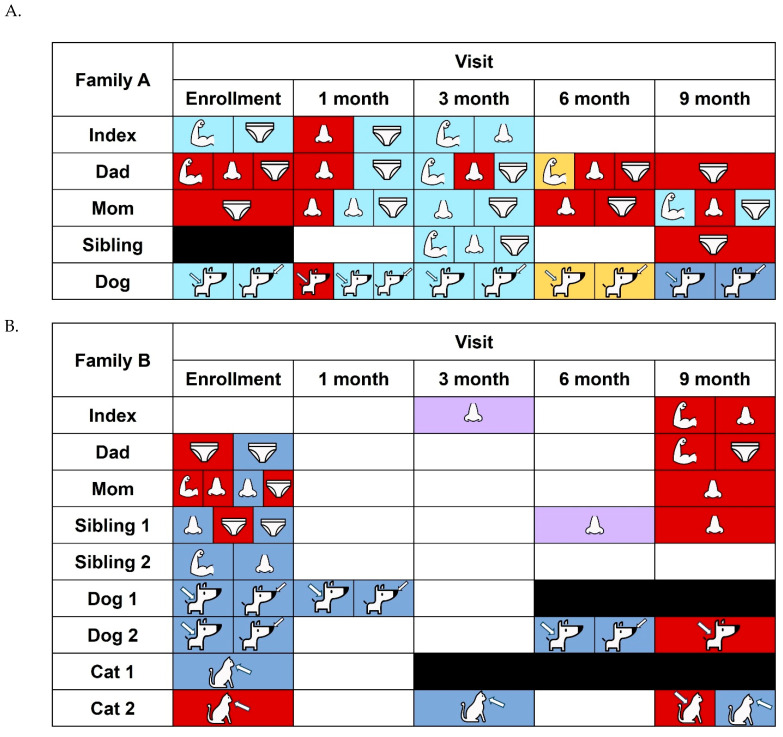

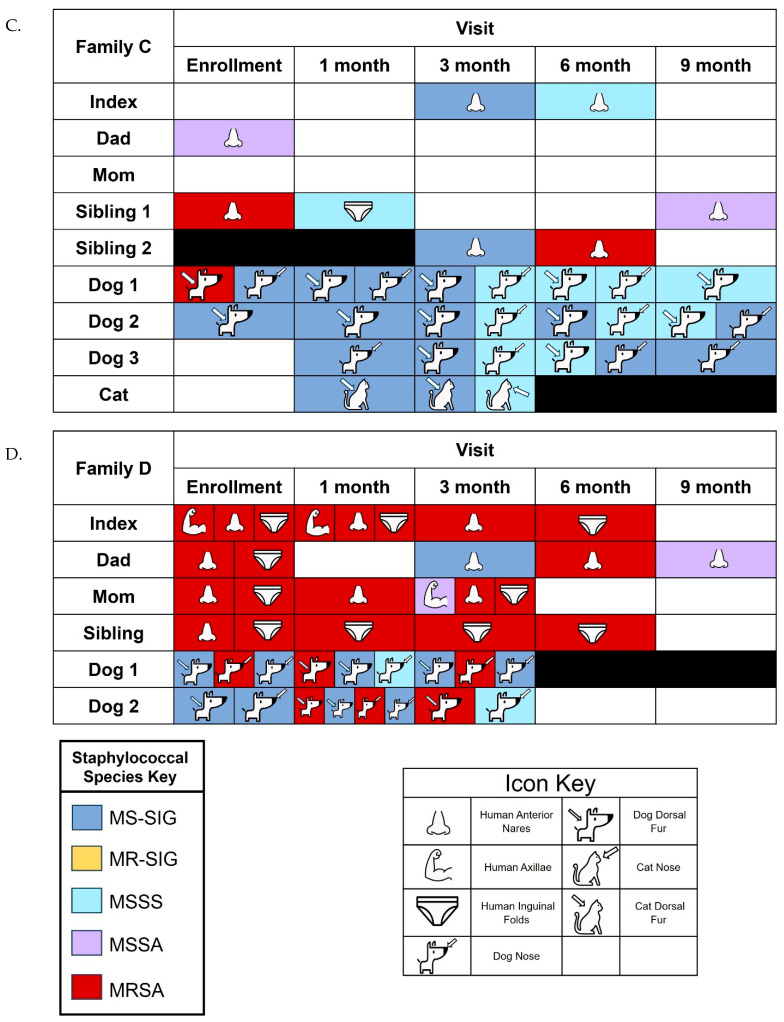
Exemplar households’ longitudinal colonization dynamics of veterinary staphylococci and *S. aureus* in humans and their pets throughout the 9-month study with color (in which each color is representative of staphylococcal species and methicillin susceptibility) and icon key. (**A**) Longitudinal colonization dynamics for Family A, which includes 4 humans and 1 dog; (**B**) longitudinal colonization dynamics for Family B, which includes 5 humans and 2 dogs and 2 cats; (**C**) longitudinal colonization dynamics for Family C, which includes 5 humans and 3 dogs and 1 cat; (**D**) longitudinal colonization dynamics for Family D, which includes 4 humans and 2 dogs. Abbreviations: MS-SIG, methicillin-susceptible *Staphylococcus intermedius* group; MR-SIG, methicillin-resistant *Staphylococcus intermedius* group; MSSS, methicillin-susceptible *Staphylococcus schleiferi*; MSSA, methicillin-susceptible *Staphylococcus aureus*; MRSA, methicillin-resistant *Staphylococcus aureus*.

The majority of humans were colonized with veterinary staphylococci at one sampling timepoint (85%), while 8% were intermittently colonized. One household experienced complex colonization patterns among pets and humans with *S. schleiferi* and SIG (Figure 3A). Co-colonization with veterinary staphylococcal species and *S. aureus* at the same timepoint occurred in 38% of humans (Table 2).

### 2.3. Colonization Concordance Within Households Between Dogs, Cats, and Humans

We evaluated colonization concordance, defined as carriage of the same staphylococcal species [35]. Of 103 dogs colonized with veterinary staphylococci, 44% lived in a household with a colonized person, and 34% were colonized with the same species as their humans at least once (Figure 3). Thirteen percent of dogs lived in a household with a colonized cat and shared one of the same species at least once (Figure 3B,C). Sixty percent of dogs lived in a household with another colonized dog and shared the same species at least once (Figure 3B–D).

All seven cats colonized with veterinary staphylococci lived in households with a colonized human and one or more colonized dogs (Figure 3B,C). Additionally, all seven colonized cats were colonized by the same species as a dog within the household. Veterinary staphylococci were not recovered from any households with only cats.

Of 39 human participants colonized with veterinary staphylococci, 35 (90%) were colonized at the same timepoint and with the same species as one or more pet within the household (Figure 3). Of the remaining four participants, three had a pet who was colonized with the same species at another sampling during the study period, while only one participant was colonized with a species different from their pet at all timepoints throughout the study.

### 2.4. Household Environmental Contamination

**Figure 4:**
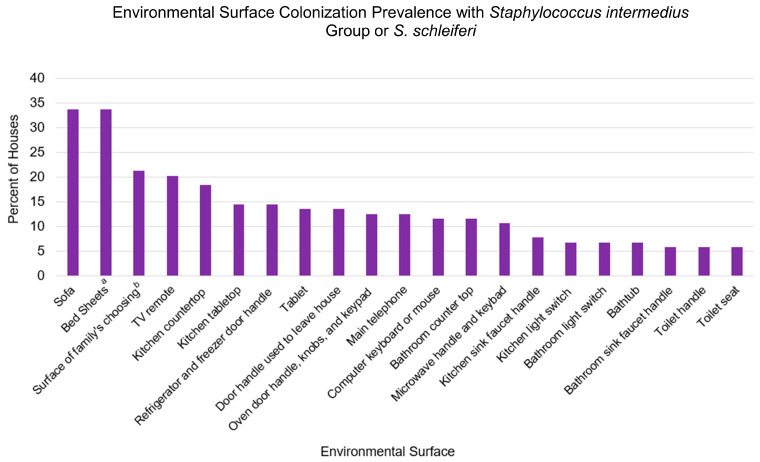
Environmental colonization prevalence with *S. intermedius* group or *S. shleiferi*. Percent of households with pets that had positive cultures for *S. intermedius* group or *S. schleiferi* at one or multiple timepoints presented for each environmental site. ^a^ Bed sheets were counted as colonized for the household if at least one set of bed sheets in the household contained veterinary staphylococci. ^b^ For “Surface of family’s choosing”, common categories included furniture (high-chairs, table surfaces, door or drawer handles), toys (stuffed animal/blanket, play pad, car/truck) and electronics (game controller, iPad, cell phone).

Veterinary staphylococci were found on environmental surfaces in 53% of pet-containing households. The median number of surfaces colonized in each household was one (range: 1–20). The sofa and bed sheets were the most commonly colonized surfaces, followed by the surface of the family’s choice, television remote control, and kitchen countertop (Figure 4). SIG was the dominant species recovered from all colonized surfaces.

### 2.5. Factors Associated with Veterinary Staphylococci Carriage

In our univariate analyses, pet type (dog or cat) was the only significant factor associated with veterinary staphylococci carriage in pets; 16% of cats were colonized compared to 71% of dogs (*p* < 0.001, Table 1). Our multivariable logistic regression model also demonstrated that dogs (pet type; odds ratio [OR] 28.22; 95% confidence interval [CI], 8.07, 145.50), as well as the burden of environmental surface contamination (number of surfaces contaminated; OR 1.72; 95% CI, 1.28, 2.52), were associated with increased odds of colonization with veterinary staphylococci (Table 3).

Univariate analyses demonstrated that humans who were primary caregivers of a colonized pet (13% vs. 0%, *p* = 0.01) or slept in a bed with a colonized pet (17% vs. 0%, *p* = 0.004) were more likely to be colonized compared to humans who were caregivers or slept in a bed with a pet who was not colonized. A binomial generalized linear mixed-effects model demonstrated that human carriage with veterinary staphylococci was associated with burden of environmental surface contamination (OR 1.27; 95% CI, 1.14, 1.41) (Table 4).

For the household environment, a logistic regression model demonstrated that household environmental contamination with veterinary staphylococci was associated with the number of pets within that household (OR 2.18; 95% CI, 1.25, 4.12) and only having dogs in the household (OR 4.88; 95% CI, 1.13 25.22) (Table 5).

### 2.6. Antimicrobial Susceptibility

Throughout the course of this study, SIG isolates were resistant to methicillin (MR-SIG) in samples collected from three (2%) dogs, no cats, and one (0.2%) human. Of these three dogs colonized with MR-SIG, one dog was not on any antibiotics, one dog was on antibiotics and had surgery prior to study enrollment, and one dog (D1) was on antibiotics prior to and during study participation and also had a chronic skin condition, skin infection, and surgery. Interestingly, the one human with MR-SIG was the primary caregiver to the dog mentioned above (D1) but had not used antibiotics prior to or during study participation. Environmental MR-SIG colonization was found in 5% (*n* = 5) of pet-containing households. Among the MR-SIG isolates recovered from pets, resistance to multiple antibiotics was common; none of the MR-SIG isolates were susceptible to trimethoprim/sulfamethoxazole (TMP-SMX), 60% were susceptible to delafloxacin, doxycycline, clindamycin, and erythromycin, and 40% were susceptible to ciprofloxacin, enrofloxacin, and marbofloxacin. Among SIG isolates susceptible to methicillin (MS-SIG), 66% of isolates recovered from pets and 88% from humans were susceptible to trimethoprim–sulfamethoxazole with high susceptibility to other tested antibiotics (Table 6). Methicillin-resistant *S. aureus* (MRSA) was recovered from 18 (12%) dogs and 7 (16%) cats.

## 3. Discussion

This investigation of coagulase-positive staphylococcal species within households demonstrates that a One Health approach is essential to understand the colonization patterns, antibiotic susceptibility, and transmission dynamics of pathogens that circulate between companion animals and human household members. This study demonstrated that SIG, *S. scheiferi*, and *S. aureus* can be shared between companion animals and humans, which can be facilitated through direct, physical contact or indirectly through environmental surfaces [28]. Additionally, we found that *S. aureus* and veterinary staphylococcal species can co-colonize the same anatomic niche in both pets and humans. These dynamics raise concerns for potential horizontal gene transfer of antimicrobial resistance and virulence genes, presenting clinical challenges for both veterinarians and physicians [3,36,37,38,39,40,41,42].

Veterinary staphylococci were recovered from the majority of dogs in this study (76%), which is at the higher end of prior prevalence reports among healthy dogs (14–73%) [37,43,44,45,46]. Colonization has also been reported to be high among dogs with pyoderma and/or otitis, (64%) [47]. The prevalence of veterinary staphylococci colonization among cats in the present study, 17%, was also higher than the previously reported 1–3% in healthy cats, but lower than colonization prevalence in cats with dermatitis or otitis (26–33%) [15,48,49]. Similar to the present study, prior research among households in which a human experienced an MRSA infection have demonstrated that 41–53% of dogs and 2–4% of cats were colonized with *S. pseudintermedius*. The higher prevalence of veterinary staphylococci in our study may reflect our longitudinal, household-based sampling approach and the sampling of multiple anatomical sites.

Consistent with prior research, dogs were more frequently colonized with veterinary staphylococcal species, compared to cats [42,50]. Indeed, cats were only colonized with veterinary staphylococci if they lived with a colonized dog or human. Additionally, the majority of dogs in our study had prolonged carriage of veterinary staphylococci, and colonization among multiple dogs within a household was common. The observation that dogs experienced sustained carriage, while most humans were only colonized at one timepoint, supports the tenet that dogs are the primary reservoir of veterinary staphylococci, cats may be secondary reservoirs, and humans become transiently contaminated by their pets [3,42,43].

Prior studies of veterinary staphylococci colonization in humans living with pets have demonstrated a range of colonization prevalence between 2 and 40% [37,43,45]. The present study found that 9% of humans living in households with pets were colonized with veterinary staphylococci. Importantly, 90% of humans were colonized with the same species at the same timepoint as their pets, suggesting the potential for interspecies transmission, consistent with prior reports [30,34,51].

Household environmental surface contamination with veterinary staphylococci was present in over 50% of pet-containing households. Previous research has identified that staphylococcal species can survive in the environment for a prolonged time [3,52,53]. In prior studies of households with methicillin-resistant *S. pseudintermedius* (MRSP)-positive pets, the environmental surfaces most commonly contaminated with veterinary staphylococci were those in close contact with the pet, including the pet bed, feeding bowls, and floor [11,29,30]. Interestingly, in the present study, veterinary staphylococci was most commonly recovered from the sofa and bed sheets, the two fabric items that were sampled and those likely in frequent contact with pets. This aligns with previous research demonstrating that cloth fabrics and soft furnishings are relevant fomites for bacteria, including *Staphylococcus aureus* [24,54,55]. Importantly, surfaces not in close contact with pets (e.g., toilet seat and sink faucet handles) were not commonly contaminated with veterinary staphylococci. Our data demonstrate that environmental surfaces may facilitate transmission of veterinary staphylococci between humans and pets, particularly as a higher number of contaminated environmental sites was associated with colonization in humans and pets. Thus, in households experiencing recurrent infections, effective infection prevention may rely on integrated interventions that simultaneously target multiple reservoirs, including household environmental surfaces, rather than solely the infected human or animal [56].

The emerging prevalence of methicillin resistance in veterinary staphylococcal species poses challenges to treating infections caused by these pathogens, particularly as these methicillin-resistant strains are often resistant to other antibiotics [10]. The prevalence of methicillin resistance (MR) in SIG and *S. schleiferi* isolates among our healthy population was low, similar to previous studies of healthy pets and those living with a human with an MRSA infection, ranging from 0 to 6% [15,44,46,57]. In contrast, among companion animals presenting for care to veterinary clinics for pyoderma and otitis, the prevalence of MRSP colonization is higher (up to 14%) [47]. We found no cats colonized with MR-SIG, similar to previous reports [48,57]. MR-SIG human colonization (0.2%) was lower than previous reports of 4.5% colonization prevalence in humans with healthy dogs. The low prevalence of methicillin resistance in our study may reflect the absence of recent veterinary antimicrobial exposure in the majority of households, as well as a primarily healthy pet population, where skin conditions (9%), recent surgery (8.5%) and recent antibiotics usage (16%) among the pets were relatively uncommon.

Veterinary staphylococcal species can cause SSTI in humans that appear indistinguishable from *S. aureus*. Among our cohort, a low proportion of SIG isolates were susceptible to trimethoprim–sulfamethoxazole (TMP-SMX), an antibiotic that is frequently prescribed empirically to humans with SSTI for presumed *S. aureus* infection, before the microbiologic etiology is known. A similarly low susceptibility to TMP-SMX was reported in SIG isolates recovered from human infections by Yarbrough and colleagues [10]. Thus, among individuals with pet exposure, careful consideration of empiric antibiotic selection for SSTI should be taken, particularly if a patient is not improving as expected [58,59].

This study has numerous strengths, including multiple samplings over time, illuminating longitudinal patterns of colonization with veterinary staphylococci and *S. aureus* in pets and humans. Additionally, we performed comprehensive environmental surface sampling to inform potential decolonization targets to mitigate transmission. This study is limited in that data were obtained from one geographical region, limiting generalizability. This study was a sub-analysis of a clinical trial conducted among households affected by *S. aureus*. While the presence of *S. aureus* did not preclude colonization with veterinary staphylococci, the colonization dynamics may differ among companion animals and humans in households not affected by *S. aureus*. Moreover, participation in the SHINE trial included a baseline decolonization regimen, followed by a 3-month randomized intervention comprising personal and/or environmental decolonization, with the goal of reducing or eliminating *S. aureus* colonization, which may have influenced longitudinal veterinary staphylococci colonization patterns. Lastly, while the comprehensive sampling facilitated detection of concordant staphylococcal species colonization among pets, humans, and their household environments, genomic sequencing was not performed on these isolates, thereby precluding evaluation at the strain level.

In this investigation of coagulase-positive staphylococci epidemiology among pets and humans in households affected by *S. aureus* infections, we have demonstrated that colonization with veterinary staphylococci is prevalent in both pets and their owners. Temporal colonization with the same staphylococcal species between pets and owners suggests interspecies anthropozoonosis transmission. As veterinary staphylococci can cause infections in humans and *S. aureus* can cause infections in companion animals, in clinical settings it is essential to inquire about the health of pets when evaluating human patients, and the health of household humans when evaluating veterinary patients. This study highlights the importance of the household and pet dynamic in consideration of the infecting pathogen and optimal antimicrobial management.

## 4. Methods

### 4.1. Study Population

Pediatric patients diagnosed with a community-onset *S. aureus* SSTI were recruited from St. Louis Children’s Hospital and community pediatric practices in metropolitan St. Louis to participate in the “*Staph* Hygiene Intervention for Eradication (SHINE)” trial between 2015 and 2021 [56]. Children with healthcare-related risk factors, nosocomial infections, and immunodeficiency were excluded from participation [60]. The household contacts and indoor companion animals (hereafter referred to as “pets”) of these index patients were also enrolled. Household contacts were defined as individuals who slept in the home for at least 4 nights per week, with no additional exclusion criteria. The Washington University Institutional Review Board and Institutional Animal Care and Use Committee approved study procedures. Written, informed consent, and assent when appropriate, were obtained for all household members (by the participant and/or guardian) and pets (by the primary caretaker).

### 4.2. Data and Specimen Collection

After the baseline enrollment visit, longitudinal visits occurred at 1, 3, 6, and 9 months. Each visit occurred at the participant’s home, where surveys were given to each household member and colonization swabs (Eswab, Becton Dickinson [BD], Franklin Lakes, NJ, USA) were collected. Colonization swabs were obtained from pets at two anatomical sites (mouth and dorsal fur) [38], from humans at three anatomical sites (nares, axillae, and inguinal folds), and from 21 household environmental surfaces and objects. Human surveys focused on health, hygiene practices, school and/or occupation, and activities within and outside of the household. Pet surveys recorded characteristics such as pet type, hair length, health, and daycare attendance or recent boarding. Household surveys recorded characteristics of the dwelling and cleaning practices.

### 4.3. Specimen Processing

For each swab specimen, 100 µL of eluent was inoculated into tryptic soy broth (TSB) with 6.5% NaCl (BBL, BD) and incubated overnight at 35 °C. TSB specimens were subsequently plated to trypticase soy agar with 5% sheep blood (blood agar plates [BAP]; BBL, BD). Colonies suspected to be veterinary staphylococci (based on colony morphology and Gram-staining) and all *S. aureus* isolates recovered from pets were identified by MALDI-TOF MS using the VITEK MS v2.0. Isolates confirmed to be a SIG species, *S. schleiferi*, or *S. aureus* by MALDI-TOF MS underwent antibiotic susceptibility testing via disk diffusion on Mueller–Hinton agar (BBL, BD) according to Clinical and Laboratory Standards Institute (CLSI) procedures [61]. For SIG species and *S. schleiferi*, the tested antibiotics included oxacillin (as a surrogate for methicillin resistance), delafloxacin, ciprofloxacin, enrofloxacin, marbofloxacin, doxycycline, omadacycline, trimethoprim–sulfamethoxazole, chloramphenicol, clindamycin, erythromycin, and mupirocin [61,62,63]. For *S. aureus* isolates, cefoxitin served as the surrogate for methicillin-resistance testing [64]. In accordance with CLSI guidelines for creating a cumulative antibiogram report, the first isolate of each staphylococcal species that was recovered from each household object or each body site of participants (pet or human) was included in the analysis [65].

### 4.4. SHINE Trial Randomization and Intervention 

As part of the SHINE trial, participants underwent a 5-day baseline decolonization regimen after enrollment, which included twice-daily intranasal mupirocin application and washing daily with chlorhexidine gluconate [56]. Households were then randomized to 1 of 3 treatment groups to be performed for 3 months: (1) Periodic Personal Decolonization Approach (“Periodic-Personal”): Each month participants applied mupirocin ointment twice a day to their anterior nares for five consecutive days, in addition to washing with chlorhexidine twice a week. (2) Environmental Hygiene Approach (“Environmental-Hygiene”): Households performed an enhanced cleaning routine once a week in addition to their additional cleaning. Specifically, participants wiped down select surfaces in the bathroom and kitchen as well as commonly used electronics with Clorox Disinfecting Wipes, washed bed linens, and changed out the kitchen sponge; they also replaced kitchen and bathroom towels daily or used disposable paper towels. (3) Integrated Approach of Personal and Environmental Hygiene Approaches (“Integrated-Approach”): Participants were asked to complete the protocols described for the Periodic-Personal and Environmental-Hygiene groups. Mupirocin and chlorhexidine were only applied to humans and not pets.

### 4.5. Statistical Analyses

R Studio (version 2025.09.2 + 418) was used to conduct all analyses. To understand factors associated with human and pet colonization with veterinary staphylococci, univariate analyses were conducted using Pearson’s Chi-square test for categorical data and Mann–Whitney U tests for continuous data. Statistical significance for univariate analysis was set at *p* < 0.05. A binomial generalized linear mixed-effects model with a fixed effect of household was used to determine factors associated with human colonization. Logistic regression models were used to discern factors associated with pet colonization and household environmental surface contamination with veterinary staphylococci (variables listed in Table 3, Table 4 and Table 5). Covariates were selected *a priori* for all models. Models were assessed using fit statistics (Akaike information criterion) and evaluation of residuals.

## Figures and Tables

**Table 1:** Pet and Human Characteristics by Veterinary Staphylococci Colonization Status.

**Pet Variables**	**Total** **N = 178 (%)**	**Colonized with Veterinary** **Staphylococci** **N = 110 (%)**	**Not Colonized with Veterinary** **Staphylococci** **N = 68 (%)**
**Pet type ^a^**			
** Cat**	**42 (24%)**	**7 (6%)**	**35 (51%)**
** Dog**	**136 (76%)**	**103 (94%)**	**33 (49%)**
Age, years, median (range)	5 (0.1–18)	5 (0.2–18)	5 (0.1–16)
Pet hair			
Long	69 (39%)	46 (42%)	23 (34%)
Short	109 (61%)	64 (58%)	45 (66%)
Primarily indoor vs. outdoor			
Indoors	174 (98%)	107 (97%)	67 (99%)
Outdoors	4 (2%)	3 (3%)	1 (1%)
Sleeps in household member’s bed	77 (43%)	41 (37%)	36 (53%)
Attends daycare	3 (2%)	2 (2%)	1 (1%)
Skin condition	17 (10%)	13 (12%)	4 (6%)
Surgery in prior 6 months	16 (9%)	9 (8%)	7 (10%)
Antibiotic use in prior 6 months	29 (16%)	20 (18%)	9 (13%)
**Human Variables**	**Total** **N = 459 (%)**	**Colonized with Veterinary** **Staphylococci** **N = 39 (%)**	**Not Colonized with Veterinary** **Staphylococci** **N = 420 (%)**
Age, years, median (range)	16 (0–75)	11 (0–46)	16 (0–75)
Female sex	255 (56%)	20 (51%)	235 (56%)
SSTI in year prior to enrollment	171 (37%)	16 (41%)	155 (37%)
Skin condition	85 (19%)	6 (15%)	79 (19%)
Antibiotic use in year prior to enrollment	257 (56%)	25 (64%)	232 (55%)
Primary caregiver to pet	146 (32%)	12 (31%)	134 (32%)
Sleeps in a bed with pet	114 (25%)	10 (26%)	104 (25%)

Bold values indicate a statistically significant difference. ^a^ Dogs were more likely to be colonized with veterinary staphylococci compared to cats, *p* < 0.001.

**Table 2:** Co-colonization with veterinary staphylococci and *S. aureus* in humans and pets.

Group ^a^	Number of Humans	Number of Dogs ^b^
MS-SIG & MSSA	4	11
MS-SIG & MRSA	5	6
MR-SIG & MRSA	1	0
MSSS & MSSA	1	0
MSSS & MRSA	4	2

Abbreviations: MS-SIG, methicillin-susceptible *Staphylococcus intermedius* group; MR-SIG, methicillin-resistant *Staphylococcus intermedius* group; MSSS, methicillin-susceptible *Staphylococcus schleiferi*; MSSA, methicillin-susceptible *Staphylococcus aureus*; MRSA, methicillin-resistant *Staphylococcus aureus.* ^a^ No humans or dogs were co-colonized with MR-SIG and MSSA. ^b^ No cats were co-colonized with veterinary staphylococci and *S. aureus*.

**Table 3:** Results of logistic regression model predicting pet colonization with veterinary staphylococci.

Covariates	OR (95% CI)
Number of Pets in Household	1.11 (0.75–1.65)
Dog vs. (Cat)	**28.22 (8.07–145.50)**
Number of Contaminated Household Environment Surfaces	**1.72 (1.28–2.52)**
Living with Colonized Human(s)	2.61 (0.71–11.91)

Bold values indicate a statistically significant odds ratio. Abbreviations: OR, odds ratio; CI, confidence interval.

**Table 4:** Results of binomial generalized linear mixed-effects models predicting human colonization with veterinary staphylococci.

Covariates	OR (95% CI)
Number of Pets in Household	1.45 (0.96–2.18)
Pet Sleeps in Bed	0.92 (0.28–2.99)
Living with a Colonized Pet	5.46 (0.63–46.88)
Being a Primary Caretaker of a Pet	0.93 (0.38–2.26)
Number of Contaminated Household Environment Surfaces	**1.27 (1.14–1.40)**

Bold values indicate a statistically significant odds ratio. Abbreviations: CI, confidence interval; OR, odds ratio.

**Table 5:** Results of logistic regression model predicting household environmental surface contamination with veterinary staphylococci.

Covariates	OR (95% CI)
Number of Pets in Household	**2.18 (1.25–4.12)**
Crowding (humans/bedrooms)	0.46 (0.14–1.37)
Cat-only Household (vs Dog-only household; Dog and Cat Household)	0.24 (0.01–2.22)
Dog-only Household (vs Cat-only household; Dog and Cat Household)	**4.88 (1.13–25.22)**

Bold values indicate a statistically significant odds ratio. Abbreviations: CI, confidence interval; OR, odds ratio.

**Table 6:** Antimicrobial susceptibility profile of *S. intermedius* group and *S. schleiferi* isolates among humans and pets.

Isolates Recovered from Pets													
Organism	Number of Isolates Tested	% Susceptible											
		OXA	DLX	CIP	ENR	MBF	DOX	OMD	TMP/ SMX	CHL	CLI	ERY	MUP
SIG ^a^	183	97	99	95	95	95	90	100	64	97	89	89	100
MS-SIG	178	100	100	97	97	97	91	100	66	97	89	89	100
MR-SIG	5	0	60	40	40	40	60	100	0	100	60	60	100
*S. schleiferi*	14	100	100	86	86	86	100	100	100	100	100	100	100
**Isolates Recovered from Humans**													
SIG	41	98	100	98	98	98	98	100	85	100	95	95	100
MS-SIG	40	100	100	100	100	100	100	100	88	100	98	98	100
MR-SIG	1	0	100	0	0	0	0	100	0	100	0	0	100
*S. schleiferi*	15	100	100	20	20	20	100	100	100	100	100	100	100

Susceptibility reported for first isolate recovered per anatomic site of each human or pet participant. Green indicates that 85–100% of isolates were susceptible; yellow indicates that 60–84% of isolates were susceptible; red indicates that 0–59% of isolates were susceptible. Abbreviations: OXA, oxacillin; DLX, delafloxacin; CIP, ciprofloxacin; ENR, enrofloxacin; MBF, marbofloxacin; DOX, doxycycline; OMD, omadacycline; TMP/SMX, trimethoprim–sulfamethoxazole; CHL, chloramphenicol; CLI, clindamycin; ERY, erythromycin; MUP, mupirocin; SIG, *Staphylococcus intermedius* group; MS, methicillin-susceptible; MR, methicillin-resistant. ^a^ Two SIG isolates recovered from pets were identified by MALDI-TOF as *S. delphini*, of which all were susceptible to all tested antibiotics.

## Data Availability

Detailed subject-level data will not be publicly available. Additional summary data may be made available upon reasonable request.
